# Stressful events, social health issues and psychological distress in Aboriginal women having a baby in South Australia: implications for antenatal care

**DOI:** 10.1186/s12884-016-0867-2

**Published:** 2016-04-26

**Authors:** Donna Weetra, Karen Glover, Mary Buckskin, Jackie Ah Kit, Cathy Leane, Amanda Mitchell, Deanna Stuart-Butler, May Turner, Jane Yelland, Deirdre Gartland, Stephanie J Brown

**Affiliations:** Healthy Mothers Healthy Families Research Group, Murdoch Childrens Research Institute, Flemington Road, Parkville, VIC 3052 Australia; South Australian Health and Medical Research Institute, North Terrace, Adelaide, 5000 Australia; Aboriginal Health Council of South Australia Inc, 220 Franklin Street, Adelaide, South Australia 5000 Australia; Women’s and Children’s Health Network, 295 South Terrace, Adelaide, South Australia 5000 Australia; Council of Aboriginal Elders Inc, 50-60 Sussex St, North Adelaide, South Australia 5006 Australia; General Practice and Primary Health Care Academic Centre, The University of Melbourne, Parkville, VIC 3052 Australia; School of Population and Global Health, General Practice and Primary Health Care Academic Centre, The University of Melbourne, Parkville, VIC 3052 Australia

## Abstract

**Background:**

Around 6 % of births in Australia are to Aboriginal and Torres Strait Islander families. Aboriginal and Torres Strait Islander women are 2–3 times more likely to experience adverse maternal and perinatal outcomes than non-Aboriginal women in Australia.

**Methods:**

Population-based study of mothers of Aboriginal babies born in South Australia, July 2011 to June 2013.

Mothers completed a structured questionnaire at a mean of 7 months postpartum. The questionnaire included measures of stressful events and social health issues during pregnancy and maternal psychological distress assessed using the Kessler-5 scale.

**Results:**

Three hundred forty-four women took part in the study, with a mean age of 25 years (range 15–43). Over half (56.1 %) experienced three or more social health issues during pregnancy; one in four (27 %) experienced 5–12 issues. The six most commonly reported issues were: being upset by family arguments (55 %), housing problems (43 %), family member/friend passing away (41 %), being scared by others people’s behavior (31 %), being pestered for money (31 %) and having to leave home because of family arguments (27 %). More than a third of women reporting three or more social health issues in pregnancy experienced high/very high postpartum psychological distress (35.6 % versus 11.1 % of women reporting no issues in pregnancy, Adjusted Odds Ratio = 5.4, 95 % confidence interval 1.9–14.9).

**Conclusions:**

The findings highlight unacceptably high rates of social health issues affecting Aboriginal women and families during pregnancy and high levels of associated postpartum psychological distress. In order to improve Aboriginal maternal and child health outcomes, there is an urgent need to combine high quality clinical care with a public health approach that gives priority to addressing modifiable social risk factors for poor health outcomes.

**Electronic supplementary material:**

The online version of this article (doi:10.1186/s12884-016-0867-2) contains supplementary material, which is available to authorized users.

## Background

Disparities in health outcomes for Aboriginal and Torres Strait Islander Australians have a long history. As a legacy of colonisation, Aboriginal and Torres Strait Islanders have been dispossessed of land and culture in ways that have profound implications for health and well-being [1]. Aboriginal Australians are more likely to experience poor physical and mental health; have higher rates of suicide, self-harm and substance abuse; and experience earlier onset of chronic disease and associated mortality compared with other Australians [2]. National data show that over half of Aboriginal first time mothers are under 20 years of age [3]. Despite the younger age at which Aboriginal women commence childbearing, they experience higher rates of medical complications in pregnancy [4], and are two to five times more likely to die from pregnancy related complications as non-Aboriginal women [5]. Aboriginal women also experience higher rates of perinatal deaths compared with non-Aboriginal women (17.1 versus 8.8 per 1000 births), and are twice as likely to have a preterm birth (13.5 % versus 8.0 %) and/or baby with a low birthweight (12.0 % versus 6.0 %) [3].

It is estimated that around 6 % of births in Australia are to Aboriginal and Torres Strait Islander families [3]. Individual states and territories within Australia have adopted different policy and service approaches to addressing disparities in Aboriginal maternal and child health outcomes. In South Australia, the state government has implemented the Aboriginal Family Birthing Program (AFBP) building on the success of two small scale regional birthing programs established in the early 1990s [6]. Scaling up of the program commenced in 2009 and it currently operates in six regional areas and in the capital city of Adelaide. Women participating in the program are cared for by Aboriginal Maternal Infant Care (AMIC) workers that work in partnership with midwives and doctors to provide antenatal, intrapartum and early postnatal care up to 8 weeks after the birth. The Aboriginal Family Birthing Program services aim to provide culturally responsive holistic care that combines high quality clinical care with attention to broader social determinants of health, consistent with the principles of comprehensive primary health care [7]. As South Australia covers a geographic area four times the size of the UK, one of the major challenges for the program is how to provide continuity of care for Aboriginal women living in regional or remote areas of South Australia needing to access specialist services only available in the major capital city of Adelaide.

This paper reports data from a population-based study, called the Aboriginal Families Study, which investigated the views and experiences of mothers having an Aboriginal baby in South Australia between July 2011 and June 2013. The study was conducted in partnership with the Aboriginal Health Council of South Australia and was designed in part to assess the views and experiences of Aboriginal women accessing different models of maternity care, including the Aboriginal Family Birthing Program services. An earlier paper from the study focusing on antenatal care compares the experiences of women attending Aboriginal Family Birthing Program services with those of women attending mainstream public maternity services [8]. In addition to assessing women’s views and experiences in different models of care, the study aimed to investigate stressful events and social health issues affecting women’s health in pregnancy and the first 6 months after giving birth. The aims of this paper are to: (i) describe the frequency and types of stressful events and social health issues experienced by Aboriginal women and families during pregnancy, (ii) to examine the relationship between the number and types of stressful events and social health issues experienced by families during pregnancy and maternal postpartum psychological distress, and (iii) consider implications for policy and health services.

## Method

The Aboriginal Families Study was conducted as a partnership between academic researchers at the Murdoch Childrens Research Institute, the University of Adelaide and the Aboriginal Health Council of South Australia Inc. The first stage of the study involved community consultations undertaken in urban, regional and remote areas of South Australia in 2008-2009 [9]. An Aboriginal Advisory Group has guided the development and conduct of the study since its inception in 2007. The community consultations highlighted the importance of women’s ‘social and emotional well-being’, and ‘knowing what’s happening in women’s lives during pregnancy’. Based on feedback from the community consultations, it was decided that to include questions in the study that: (i) asked about stressful events and social health issues experienced by Aboriginal women and families in pregnancy, (ii) inquired about women’s social and emotional well-being, and (iii) asked women about what helped them to stay ‘positive and strong’.

### Study population

Women were recruited to the study via public maternity hospitals, Aboriginal community controlled health services, community organisations and community events, the interviewers’ own community networks, and through study participants referring women to the study. Information about the study was circulated via community newsletters, information brochures and posters in community agencies, and by community radio. To be eligible to take part women needed to be aged ≥ 14 years, and to have given birth to an Aboriginal baby in the study period. Women consenting to participate were invited to complete a structured interview when their baby was between 4 to 12 months old. A team of 12 Aboriginal research interviewers and one non-Aboriginal research interviewer recruited eligible women living in urban, regional and remote areas of South Australia over a 2-year period. Interviewers were based in Adelaide and in regional areas, including Port Augusta, Murray Bridge and Port Lincoln. Interviewers made regular visits to other communities, including Ceduna, Yalata, Whyalla, Coober Pedy and Mount Gambier.

Women recruited via public hospitals were mostly approached on postnatal wards in the first few days after giving birth. Aboriginal researchers made regular visits to the postnatal ward of the largest tertiary maternity hospital in Adelaide to provide information about the study to Aboriginal women and their families. This included verbal and written information about the study. Women who expressed interest in participating in the study were asked to provide contact details, and were then followed up 3–6 months later. Women recruited via other agencies, including other metropolitan and regional hospitals and health services, mostly heard about the study from staff working in the agency that gave out information about the study on our behalf. Women’s contact details were only passed on to study staff if women expressed interest in hearing more about the study. Once a community referral was received, women were initially followed up by phone, and then an appointment was made to meet with the woman either at the community based agency making the referral, or in her own home if she preferred. Women also heard about the study via community networks, and at community events. In addition, the Aboriginal researchers drew on their own community networks to distribute information and recruit women to the study.

Women were offered the option of completing the questionnaire with an interviewer or completing the questionnaire themselves. Women were able to provide written or verbal consent to participate. Women aged 14–17 were encouraged to discuss the project with another family member, parent or legal guardian before deciding to take part. However, parental or guardian consent was not a requirement for participation. After completing the questionnaire, women were given a gift voucher in appreciation of their participation. Further details about the study, including information about the community consultations that informed the study protocol and methods are available in a previous paper [9].

### Questionnaire

The 44 page questionnaire included sections which asked about: ‘you and your family’, ‘about your pregnancy care’, ‘about your life when you were pregnant’, ‘the first few months after the baby’s birth’ and ‘support for you and staying on track’. Information was collected on a range of social and maternal characteristics, including the participant’s age, educational qualifications, place of residence, and number of adults and children living in the household. Pilot testing of the questionnaire resulted in modifications to ensure that questions were acceptable to women of all ages and backgrounds.

### Measures and definitions

Assessment of stressful events and social health issues drew on items included in a measure developed for a population-based study of women giving birth in South Australia and Victoria (broadly based on the PRAMS study), with additional items incorporated based on consultations with Aboriginal community organisations and communities in South Australia and Victoria (10). In the section of the questionnaire that asked about ‘your life when you were pregnant’, women were asked to indicate whether or not 12 stressful events and social health issues had happened to them during their pregnancy. Stressful events included: death of a family member; serious illness or injury; and having to stop work or study, not because of the pregnancy. Social health issues included: housing problems, trouble with police or having to go to court; leaving home because of a family argument or fight; being scared by other people’s behaviour; being pestered for money; being pushed, shoved or assaulted; having drug and alcohol problems; and having a partner with drug and alcohol problems. Pre-given response categories were ‘yes’, ‘no’ or ‘prefer not to answer’. The final set of items included in this measure was determined in consultation with the Aboriginal Advisory Group and pilot-tested with Aboriginal women living in urban, regional and remote areas of South Australia to assess acceptability and ease of completion. As a result of piloting minor changes were made to wording of some items, and some items originally included in the pilot version were omitted from the final version. A copy of the questionnaire containing this measure is available via the study website [11].

The questionnaire also included the 5-item version of the Kessler Psychological Distress Scale (K-5) [12]. The longer 10 item (K-10) and 6 item (K-6) versions of this scale were designed to measure non-specific psychological distress encompassing both anxiety and depression, and have been extensively validated and shown to perform well in population-based studies [12–14]. The K-5 was devised for use in the National Aboriginal and Torres Strait Islander Health Survey (NATSIHS), after concerns were raised about the wording of some items in the K-6 and K-10 with respect to their acceptability to Aboriginal people [12]. The 5-item version includes the following questions: “During the past 4 weeks, about how often did you feel … (i) nervous?, (ii) without hope?, (iii) restless or jumpy?, (iv) everything was an effort?, and (v) so sad nothing could cheer you up?. Response options for each item were: ‘none of the time’, ‘a little of the time’, ‘some of the time’, ‘most of the time’ and ‘all of the time’ were scored from 1 to 5 to produce a possible range of 5-25, with higher scores indicating higher psychological distress. Scores were calculated using the framework devised for the NATSIHS, with a score of 5–7.99 categorised as low, 8–11.99 categorised as moderate, 12–14.99 categorised as high, and 15–25 categorised as very high psychological distress [12]. The K-5 was selected for use in the current study in preference to other measures of social and emotional wellbeing that have been used in Aboriginal populations such as the Edinburgh Postnatal Depression Scale [15, 16] and Strong Souls Checklist [17] based on positive feedback about the acceptability of the K-5 from women who participated in the pilot study. The K-5 questions were located towards the end of the interview booklet, and were immediately followed by questions asking whether women had someone to talk to about things happening in their lives. Training and guidelines were providing for research interviewers regarding provision of support, information and referral to local services for women identified through the research as potentially needing additional support.

### Data analysis

Quantitative data were analysed using Stata version 13 (StataCorp) [18]. We calculated the proportion of women reporting individual stressful life events and social health issues during pregnancy, and the proportions of women reporting no issues, one to two issues, and three or more issues, consistent with the approach taken in population based study of women giving birth in South Australia and Victoria conducted in 2008 [10]. Univariable logistic regression was used to assess associations between participant characteristics and (i) number of social health issues (<3/≥3), and (ii) level of psychological distress according to scores on the K-5. Results on the K-5 were dichotomised (<12/≥12) with a cut off score of ≥12 reflecting ‘caseness’ for psychological distress. Multivariable logistic regression was used to assess the relationship between number of social health issues reported (none/1-2/3 or more) and postpartum psychological distress (K-5 score of ≥12), taking into account maternal social characteristics as potential confounders. Sample size estimates reported in the study protocol were based on the primary outcome measure for the study, which is not the subject of this paper [8]. Posthoc power calculations showed that with a sample of 344, the study had 80 % power with alpha of 0.05 to detect an odds ratio of 3 for the outcome of high/very high psychological distress (K-5 score of ≥12), on the K-5 comparing women experiencing no social health issues with women reporting three or more stressful events and social health issues.

Open-ended responses to a question that asked women to comment on ‘what keeps them positive and strong’ were analysed thematically by two members of the research team (DW, SB). Any discrepancies in coding were discussed and resolved by agreement.

Ethics approval, including specific provisions for recruiting women aged 14–17 years, and offering women the option of verbal consent, was obtained from the Aboriginal Human Research Ethics Committee of South Australia, the South Australian Department of Health, the Women’s and Children’s Health Network, the Lyell McEwin Hospital, Adelaide, and the Royal Children’s Hospital, Melbourne. The study was adherent to the STROBE criteria as outlined in Additional file 1.

## Results

Of 418 women who expressed interest in the study, 348 completed the interview booklet (83 %). One woman was excluded because she had all of her pregnancy care outside South Australia, and a further three women because of incomplete consent forms. The final sample included 344 women: 178 (52 %) were interviewed by an Aboriginal research interviewer, and 166 (48 %) chose to self-complete the questionnaire. The approximate time taken to complete the questionnaire was 45 min (range 30–90 min). The average age of the index child at the time women completed the interview booklet was 7 months (SD 3, range 1–17 months).

Social characteristics of participants are shown in Table 1. Most participants (90 %) were Aboriginal, a small number of participants identified as Torres Strait Islander or both Aboriginal and Torres Strait Islander (8 women, 2 %), and 7 % were non-Aboriginal. The mean age of study participants was 25 years (SD 6, range 15–43 years), with 16 % aged under 20, and 57 % aged less than 25 consistent with the age distribution for births to Aboriginal women recorded in South Australian routinely collected perinatal data [8]. Household size ranged from one to 14 people, and the number of children in households from one to 10 children. 39 % of study participants were living in Adelaide at the time of completing the interview booklet and 61 % were living in regional or remote areas of South Australia. One in four women lived in a remote area. 52 % of study participants had completed a post-secondary qualification and over half (52.3 %) reported that they were not smoking cigarettes in pregnancy.

**Table 1:** Social characteristics of study participants stratified by frequency of stressful events and social health issues during pregnancy^a^

			Number of social health issues						
	Total		None		1-2 issues		3-11 issues		Chi2
	n	Col %	n	%	n	%	n	%	*p*-value
Mother’s age when baby born									
15–19 years	55	16.0	9	16.4	13	23.6	33	60.0	0.09
20–24 years	140	40.7	22	15.7	41	29.3	77	55.0	
25–29 years	91	26.5	10	11.0	24	26.4	57	62.6	
30+ years	58	16.9	5	8.6	27	46.6	26	44.8	
Aboriginal or Torres Strait Islander									
Aboriginal &/or Torres Strait Islander	319	92.7	45	14.1	93	29.2	181	56.7	0.09
Non-Aboriginal	25	7.3	1	4.0	12	48.0	12	48.0	
Place of residence									
Major city	134	39.0	11	8.2	40	29.9	83	61.9	0.09
Regional	123	35.8	19	15.4	43	35.0	61	49.6	
Remote	87	25.3	16	18.4	22	25.3	49	56.3	
Number of children in family									
One child	145	42.2	23	15.9	42	29.0	80	55.2	0.45
2-3 children	127	36.9	17	13.4	36	28.3	74	58.3	
4–10 children	72	20.9	6	8.3	27	37.5	39	54.2	
Total number of people (adults & children) in household									
1-2 people	19	5.8	0	0.0	6	31.6	13	68.4	0.44
3-4 people	141	42.9	21	14.9	37	26.2	83	58.9	
4-5 people	59	17.9	10	16.9	19	32.2	30	50.8	
5–14 people	110	33.4	13	11.8	37	33.6	60	54.5	
Highest educational qualification									
Less than year 12	134	39.0	20	14.9	33	24.6	81	60.4	0.011
Year 12	33	9.6	4	12.1	18	54.5	11	33.3	
Certificate/Traineeship	155	45.1	17	11.0	45	29.0	93	60.0	
Diploma/Degree	22	6.4	5	22.7	9	40.9	8	36.4	
Health care concession card									
Yes	296	87.1	36	12.2	85	28.7	175	59.1	0.006
No	44	12.9	10	22.7	19	43.2	15	34.1	
Smoked cigarettes in pregnancy									
No	175	51.6	30	17.1	64	36.6	81	46.3	0.001
Yes	164	48.4	15	9.1	40	24.4	109	66.5	
Total	344	100.0	46	13.4	105	30.5	193	56.1	

^a^Denominators vary due to missing values

In order to assess the representativeness of the sample, we compared participant characteristics with data collected by the South Australian Pregnancy Outcome Unit for all births to Aboriginal mothers in 2011 [8]. This showed that participants were largely representative in relation to maternal age, gestation and infant birthweight. Women having their first baby appeared to be slightly over-represented (42 % of participants versus 34.3 % of recorded births to Aboriginal mothers in 2011 data), and women giving birth at metropolitan hospitals slightly under-represented (53 % of participants versus 59 % of recorded births to Aboriginal mothers in 2011 data).

Just over half (56 %) of women in the study had experienced three or more stressful events and social health issues during pregnancy, and one in four (27 %) had experienced between five to 12 issues. There were no differences in reported exposure to stressful events and social health issues related to maternal age, number of children and adults in the household or place of residence. Women who reported smoking cigarettes in pregnancy and women who had a health care concession card were more likely to report three or more social health issues, and women who had completed a degree or diploma were less likely to report multiple issues.

Reported frequencies of stressful events and social health issues are shown in Table 2, stratified by maternal age. The six most commonly reported social issues were: being upset by family arguments, housing problems, family member or friend passing away, being scared by others people’s behavior, being pestered for money and having to leave home because of family arguments. There were some issues that appeared to be less common amongst younger women (e.g. problems with drugs and alcohol), and some that appeared to be less common amongst older women (e.g. problems with the police or having to go to court). However, the study had limited power to assess differences between subgroups.

**Table 2:** Frequency of stressful events and social health issues by maternal age*

			Maternal age at birth of index child								
	Total		15–19 years		20–24 years		25–29 years		30+ years		Chi^2^
	n	%	n	%	n	%	n	%	n	%	*p*-value
Upset by family arguments	187	55.3	28	51.9	72	52.6	57	64.0	30	51.7	0.29
Housing problems/moved house	144	42.6	23	43.4	66	48.5	38	41.8	17	29.3	0.10
Family member or a friend passed away	137	40.9	20	38.5	51	37.8	42	46.7	24	41.4	0.59
Pestered for money	106	31.3	12	22.2	43	31.4	27	29.7	24	42.1	0.15
Scared by other people’s behaviour	103	30.7	15	27.8	38	28.1	26	28.9	24	42.1	0.24
Left home because of a family argument	90	26.6	17	32.1	38	27.5	24	26.4	11	19.6	0.51
Very sick or badly injured (not related to pregnancy)	81	24.3	12	22.2	30	22.1	22	24.7	17	31.5	0.57
Partner had problems with drugs or alcohol	70	21.8	12	24.0	24	18.0	19	22.4	15	28.3	0.46
Had to stop working or studying (not related to pregnancy)	53	16.1	3	6.0	26	19.3	16	17.8	8	14.5	0.17
Pushed, shoved or assaulted	53	15.9	8	15.1	19	14.2	15	16.9	11	19.3	0.83
Problems with the police or need to go to court	42	12.5	6	11.1	18	13.1	14	15.7	4	7.0	0.46
Mother had problems with drugs or alcohol	30	8.9	2	3.7	16	11.7	6	6.7	6	10.7	0.27
Total			55	100.0	140	100.0	91	100.0	58	100.0	

*Denominators vary due to missing values

Table 3 reports data on psychological distress experienced by women in the study according to scores on the K-5. Seven women (2 %) had missing data on the K-5 and were excluded from further analyses. The mean score on the K-5 was 9.4, standard deviation = 3.9, range 5–25. The proportion of women in the study experiencing high to very high levels of psychological distress (K-5 score of ≥12) was 24.7 % (95 % CI 20.1–29.6) Women experiencing a greater number of stressful events and social health issues reported higher levels of psychological distress. Among women experiencing 3–12 issues during pregnancy, 35.6 % (95 % CI 28.8–42.9) reported high to very high levels of psychological distress.

**Table 3:** Level of maternal postpartum psychological distress by number of stressful events and social health issues in pregnancy (*n* = 337)^a^

Psychological			Stressful events/social health issues					
Distress (K-5)	Total		None		1-2 issues		3–11 issues	
	n	%	n	%	n	%	n	%
Low	133	39.5	25	55.6	50	48.1	58	30.9
Moderate	121	35.9	15	33.3	43	41.3	63	33.5
High	42	12.5	2	4.4	6	5.8	34	18.1
Very High	41	12.2	3	6.7	5	4.8	33	17.6

^a^Denominators vary due to missing values

The social characteristics of women reporting low/moderate psychological distress (K-5 score of <12) and high/very high psychological distress (K-5 score of ≥12) are shown in Table 4. Compared to women living in urban areas, women living in remote parts of South Australia had higher odds of scoring above 12 on the K-5. Other characteristics associated with higher scores on the K-5 (score of ≥12) were: having a health care concession card, having more than three children, not completing year 12, and smoking during pregnancy. Compared to women aged 20–24 years, younger women (under 20) and women aged 25–30 years had slightly raised odds of scoring above the cut off score of ≥12, with results only bordering on statistical significance.

**Table 4:** Relationship between maternal social characteristics and psychological distress

			Psychological Distress					
	Total		Low/Moderate		High/Very high			
	n	Col %	n	%	n	%	OR	95 % CI
Mother’s age when baby born								
15–19 years	54	16.0	37	68.5	17	31.5	1.8	[0.9,3.7]
20–24 years	135	40.1	108	80.0	27	20.0	1.0	[ref]
25–29 years	91	27.0	63	69.2	28	30.8	1.8	[1.0,3.3]
30+ years	57	16.9	46	80.7	11	19.3	1.0	[0.4,2.1]
Aboriginal or Torres Strait Islander								
Aboriginal &/or Torres Strait Islander	312	92.6	231	74.0	81	26.0	1.0	[ref]
Non-Aboriginal	25	7.4	23	92.0	2	8.0	0.2	[0.1,1.1]
Place of residence								
Major city	130	38.6	102	78.5	28	21.5	1.0	[ref]
Regional	121	35.9	96	79.3	25	20.7	0.9	[0.5,1.7]
Remote	86	25.5	56	65.1	30	34.9	2.0^*^	[1.1,3.6]
Number of children in family								
One child	141	41.8	110	78.0	31	22.0	1.0	[ref]
2-3 children	125	37.1	97	77.6	28	22.4	1.0	[0.6,1.8]
4–10 children	71	21.1	47	66.2	24	33.8	1.8	[1.0,3.4]
Total number of people (adults & children) in household								
1-2 people	18	5.6	16	88.9	2	11.1	1.0	[ref]
3-4 people	140	43.3	108	77.1	32	22.9	2.4	[0.5,10.9]
4-5 people	59	18.3	46	78.0	13	22.0	2.3	[0.5,11.1]
5–14 people	106	32.8	75	70.8	31	29.2	3.3	[0.7,15.2]
Highest educational qualification								
Less than year 12	131	38.9	83	63.4	48	36.6	2.8^***^	[1.6,4.9]
Year 12	32	9.5	23	71.9	9	28.1	1.0	[ref]
Certificate/Traineeship	152	45.1	126	82.9	26	17.1	1.9	[0.8,4.6]
Diploma/Degree	22	6.5	22	100.0	0	0.0	1.0	[1.0,1.0]
Health care concession card								
No	44	13.2	41	93.2	3	6.8	1.0	[ref]
Yes	289	86.8	211	73.0	78	27.0	5.1^**^	[1.5,16.8]
Smoked cigarettes in pregnancy								
No	171	51.2	144	84.2	27	15.8	1.0	[ref]
Yes	163	48.8	108	66.3	55	33.7	2.7^***^	[1.6,4.6]
Someone to talk to in pregnancy								
Sometimes/No/Didn’t want to talk	103	30.8	65	63.1	38	36.9	1.0	[ref]
Always	232	69.3	188	81.0	44	19.0	0.4^***^	[0.2,0.7]
Total	337	100.0	254	75.4	83	24.6		

Denominators vary due to missing values

*p < 0.05

**p < 0.01

***p < 0.001)

Table 5 shows; (i) the proportions of women experiencing specific events and social health issues in pregnancy that reported high or very high psychological distress in the postpartum period (K-5 score of ≥12), and (ii) odds ratios and 95 % confidence intervals for the association between each issue and scores above the cut-off for high to very high distress on the K-5. Five out of the 12 issues were associated with greater than three fold increases in odds of high to very high psychological distress. These were: ‘being scared by other people’s behaviour’, ‘being pushed, shoved or assaulted in pregnancy’, ‘having problems with drugs or alcohol’, ‘partner having problems with drugs or alcohol’, and ‘being upset by family arguments’.

**Table 5:** Relationship between stressful events and social health issues during pregnancy and maternal psychological distress during first 12 months postpartum (*n* = 331)

		High/very high psychological distress(K5 score ≥12)		Odds Ratio	95 % CI
	n	n	%		
Housing problems/had to move house	140	40	28.6	1.5	[0.9,2.4]
Very sick or badly hurt	78	27	34.6	1.9^*^	[1.1,3.3]
Problems with the police/needing to go to court	42	14	33.3	1.7	[0.8,3.3]
Problems with drugs or alcohol	30	15	50.0	3.6^***^	[1.7,7.8]
Partner had problems with drugs or alcohol	70	28	40.0	3.1^***^	[1.8,5.6]
Scared by other people’s behaviour	100	42	42.0	3.8^***^	[2.2,6.4]
Pestered for money	104	41	39.4	2.9^***^	[1.7,4.9]
Upset by family arguments	183	62	33.9	3.3^***^	[1.9,5.8]
Family member or a friend passed away	134	41	30.6	1.7^*^	[1.0,2.7]
Left home because of a family argument	87	31	35.6	2.2^**^	[1.3,3.7]
Had to stop working or studying	52	9	17.3	0.6	[0.3,1.4]
Pushed, shoved or assaulted	53	25	47.2	3.6^***^	[1.9,6.6]

*p < 0.05

**p < 0.01

***p < 0.001

Multivariable logistic regression analyses were undertaken to obtain a more precise estimate of the association between exposure to stressful events and social health issues during pregnancy and maternal postpartum psychological distress taking into account social characteristics. Variables included in the model shown in Table 6 were: number of stressful events and social health issues as the exposure of main interest (3 levels), maternal age, maternal education and place of residence (urban, regional, remote). These factors were selected based on associations evident in univariable analyses, and the advice of the study’s Aboriginal Advisory Group with regard to factors most salient to the community. The adjusted odds of women reporting high to very high psychological distress (K-5 score of ≥12) were five times higher among women reporting 3–12 stressful events or social health issues during pregnancy, compared with women reporting no issues. Women who had completed post-secondary education had lower odds of reporting high to very high psychological distress that remained statistically significant in the adjusted model. In contrast, women living in remote areas were more likely to report high to very high distress.

**Table 6:** Association between number of stressful events and social health issues during pregnancy and maternal postpartum psychological distress (K5 score of ≥12) (*n* = 331)

	Total	High/very high psychological distress		Unadjusted Odds Ratio	95 % CI	Adjusted Odds Ratio	95 % CI
	n	n	%				
Social health issues							
None	45	5	11.1	1.0	[ref]	1.0	[ref]
1-2 issues	104	11	10.6	0.9	[0.3,2.9]	1.1	[0.3,3.4]
3–11 issues	188	67	35.6	4.4^**^	[1.7,11.8]	5.4^**^	[1.9,14.9]
Mothers’ age when baby born							
15–19 years	54	17	31.5	1.8	[0.9,3.7]	1.6	[0.7,3.5]
20–24 years	135	27	20.0	1.0	[ref]	1.0	[ref]
25–29 years	91	28	30.8	1.8	[1.0,3.3]	2.0	[1.0,3.9]
30+ years	57	11	19.3	1.0	[0.4,2.1]	1.2	[0.5,2.9]
Maternal education							
Post-secondary education	174	26	14.9	1.0	[ref]	1.0	[ref]
Year 12 or less	163	57	35.0	3.1^***^	[1.8,5.2]	3.6^***^	[2.0,6.4]
Place of residence							
Major City	130	28	21.5	1.0	[ref]	1.0	[ref]
Regional	121	25	20.7	0.9	[0.5,1.7]	0.9	[0.5,1.8]
Remote	86	30	34.9	2.0^*^	[1.1,3.6]	1.8	[0.9,3.6]
Total	331	81	24.5				

*p < 0.05

**p < 0.01

***p < 0.001

Two-thirds of women (235/341, 69 %) said that they always had someone to talk to about things happening in their lives. One in five (72/341, 21 %) only sometimes had someone to talk to, 5 % (17/341) had no-one to talk to and 5 % (17/341) did not want to ‘talk about personal stuff’. There was a trend for older women to be more likely to have someone to talk to and younger women under 25 appeared to be more likely not to want to talk about personal things happening in their lives. Including this measure of social support in the multivariable model described above did not change the conclusion that women exposed to three or more stressful events or social health issues during pregnancy are at greater risk of postpartum psychological distress (adjusted odds ratio = 4.6, 95 % Confidence Interval 1.6–13.2).

Almost all women who took part in the study commented on what helped them to stay positive and strong. Common themes in their responses were: having happy, healthy children; the support of their families, and the support of their partners. Women also spoke about being a role model for their children, and believing in themselves and their capacity to be a ‘strong mother’. Other themes in women’s comments about what helped them stay strong included: study, education and work; life experiences; doing things to stay healthy and look after themselves; and support from services.

## Discussion

Evidence that Aboriginal families in Australia experience a disproportionate burden of social health issues is not new [19, 20]. However, to our knowledge the Aboriginal Families Studies is the first population-based study to collect and report information about the extent and nature of social health issues experienced by Aboriginal women and families *during pregnancy*.

The research highlights unacceptably high rates of housing stress affecting Aboriginal women and families. This undoubtedly has an impact on where and how women access antenatal care, and the extent to which they are able to make healthy lifestyle choices to eat well, exercise, and modify smoking and alcohol consumption during pregnancy. Many women (over 40 %) reported that they had experienced the loss of a family member or friend during their pregnancy. The strong connections in families, larger family size and shorter life expectancy of Aboriginal people make it a common experience for Aboriginal families to be grieving while preparing for the birth of a new baby. Although a very common experience for Aboriginal families, the impact of this on women’s physical and emotional wellbeing during and after pregnancy is poorly understood.

A large number of women reported experiences of family or community conflict. One in three had been scared by other people’s behavior while they were pregnant, one in five had left home because of a family argument, and one in six had been physically assaulted. There is an accumulating evidence of the negative impact of family violence and other kinds of social adversity on rates of miscarriage, preterm birth and low birthweight, as well as other long term health consequences for women and children [21–27].

One in four women in the study (24.7 %) reported high to very high psychological distress in the postpartum period. This is markedly higher than estimates of maternal psychological distress among general population samples [28, 29], and estimates using the same measure (K-5) to assess psychological distress among similar aged non-Aboriginal Australian women [30]. While attributing causation is problematic in observational studies, the 2-3-fold increase in odds of high to very high psychological distress associated with individual social health issues and stressful events in pregnancy, and the evidence of cumulative impact of multiple social health issues (5-fold increase in odds for women experiencing three or more social health issues) are strongly suggestive of a causal relationship.

When we were designing the study, community members told us it was important for the study to collect information on these issues, and for this information to be used to inform changes to services to improve outcomes for Aboriginal families. A major strength of the study is the partnership with the Aboriginal Health Council of South Australia, and the processes that were used to engage Aboriginal community organisations and communities throughout the planning and evolution of the study [9]. The Aboriginal Advisory Group worked closely with the research team to guide the way that the interviewers worked with communities and families, and to assist with interpreting the findings. Approximately a quarter of all Aboriginal women who gave birth in South Australia over a 2-year period took part in the study. The high level of participation in regional and remote communities, and engagement of so many younger women, is a testament to the Aboriginal research interviewers’ skills working with communities across South Australia. It is extremely rare in epidemiological studies to achieve a population-based sample that is representative in relation to maternal age. This feature of the sample greatly strengthens the generalisability of the findings.

Notwithstanding these strengths, the study also has a number of limitations. The K-5, while highly acceptable to participants, has not been validated against diagnostic interview in an Indigenous population. No interviews were conducted with fathers, or with other family members. This was a decision based on funding constraints. Although the interviewers travelled to many areas of South Australia, we were unable to visit all regions of the state. Other limitations include the decisions not to collect data on income, relationship status, intimate partner abuse or alcohol use in pregnancy: all of which were seen as potentially intrusive questions for the interviewers to ask, that may have resulted in lower overall participation. While it is not possible to determine whether this would have been the case, respecting community advice was an important principle underpinning the way that the researchers and the Aboriginal Health Council of South Australia worked together to undertake the study. The questions that asked about stressful events and social health issues and symptoms of psychological distress might also be considered sensitive. The fact that these questions were so well answered, with very few women opting to skip individual items, demonstrates the benefits of engaging Aboriginal communities in the design of research, and of careful pre-testing of study instruments and study methods. The role of the small team of Aboriginal interviewers in facilitating women’s involvement in the study is also an important aspect of the way the study was conducted and likely to have contributed to the low level of missing data. Other studies using the K-10 and K-5 with Aboriginal and Torres Strait Islander populations, but not involving the same degree of community consultation and engagement or involvement of Aboriginal researchers in data collection, have reported higher levels of missing data (>14 % for the K-5, and 16 % for the K-10) [30, 31].

Women were very open about things that were happening in their lives, and the high level of stressful events and social health issues in urban, regional and remote communities shocked even members of the Aboriginal Advisory Group. Non-Aboriginal women in Australia are much less likely to experience cumulative stresses and social health issues in pregnancy. Eighteen percent of non Aboriginal women participating in a population-based survey of women giving birth in South Australia and Victoria in 2008 reported three or more stressful events and social health issues, compared with 56 % of women in the Aboriginal Families Study [10, 28]. Acting on this information is a priority. Women themselves identified that what helped them stay healthy and strong during pregnancy was their children, families and community. Antenatal care provides a window of opportunity to support women and families coping with multiple social health issues. However, public maternity services are often under resourced and lack systems to address social determinants of poor maternal and child health outcomes [10]. In order to improve outcomes, there is a pressing need to re-frame current models of care to combine high quality clinical care with a public health approach that gives priority to addressing modifiable social health risk factors for poor health outcomes [32–34].

Central to this is the need for better integration of systems to support women and families experiencing housing problems, family violence, problems with drugs and alcohol and other social health issues as a core component of pregnancy care. There is evidence that strengthening cross-sector collaboration and multi-disciplinary team-based approaches to antenatal care, involving Aboriginal primary care and community controlled health services in flexible program delivery, providing outreach and transport, and tailoring programs to local community needs are likely to improve maternal and child health outcomes [35–39].

However, few programs and initiatives specifically designed to address the needs of Aboriginal women and families have been subject to rigorous evaluation, limiting conclusions that can be drawn from these studies [39, 40].

Looking beyond this literature, there is increasing recognition of the need for maternity services in high income countries to take steps to address the needs of disadvantaged and vulnerable populations, such as families of refugee background, women experiencing family violence, adolescent mothers and women with substance abuse problems [41–45]. A systematic review of RCTs and observational studies examining antenatal care programs focusing on disadvantaged populations in high income countries found limited evidence of improved perinatal outcomes, but noted a number of ‘promising’ intervention strategies requiring further evaluation [46]. These include: group antenatal care, multi-faceted antenatal care programs targeting a range of risk factors for poor outcomes, and financial incentives for health services to tailor care to specific populations [46]. Few studies have examined the impact of antenatal programs on a broader range of longer term maternal and child health outcomes in disadvantaged populations.

## Conclusion

Our findings highlight unacceptably high rates of social health issues affecting Aboriginal women and families during pregnancy, and high levels of associated postpartum psychological distress. In order to improve Aboriginal maternal and child health outcomes, there is an urgent need to combine high quality clinical care with a public health approach to antenatal care that gives priority to addressing modifiable social risk factors for poor maternal and child health outcomes. Attention to the circumstances of women’s lives—including housing problems, family violence, drug and alcohol problems and other consequences of social disadvantage—should be an integral and core component of antenatal care for Aboriginal families and other socially disadvantaged populations. Implementation studies with longer term follow up of mothers and children are needed to refine approaches, and demonstrate benefits.

## Additional file

Additional file 1: STROBE Statement—checklist of items that should be included in reports of observational studies. (DOC 85 kb)

## References

[1] Australian Government. Aboriginal Torres Strait Islander Health Plan 2013-2023. http://www.health.gov.au/natsihp (Accessed April 2016)

[2] Vos T, Barker B, Begg S, Stanley L, Lopez AD (2009). Burden of disease and injury in Aboriginal and Torres Strait Islander Peoples: the Indigenous health gap. Int J Epidemiol.

[3] Li Z, Zeki R, Hilder L, Sullivan EA (2012). Australia's mothers and babies 2010. Perinatal statistics series no. 27. Cat. no. PER 57.

[4] Eades S, Read AW, Stanley FJ, Eades FN, McCaullay D, Williamson A (2008). Bibbulung Gnarneep (‘solid kid’): causal pathways to poor birth outcomes in an urban Aboriginal birth cohort. J Paediatr Child Health.

[5] Sullivan EA, Hall B, King JF (2007). Maternal deaths in Australia 2003–2005. Maternal deaths series no. 3. Cat. no. PER 42.

[6] Stamp G, Champion S, Anderson G, Warren B, Stuart-Butler D, Doolan J, Boles C, Callaghan L, Foale A, Muyambi C. Aboriginal maternal and infant care workers: partners in caring for Aboriginal mothers and babies. Rural Remote Health. 2008;8:883. 18656994

[7] Middleton P, Glover K, Weetra D, Bubner T, Crowther C, Rumbold A, Brown SJ. Evaluation of the Aboriginal Family Birthing Program. Adelaide: RANZCOG 2014 Indigenous women’s health meeting; 2014.

[8] Brown SJ, Weetra D, Glover K, Buckskin M, Ah Kit J, Leane C, Mitchell A, Stuart-Butler D, Turner M, Gartland D, Yelland J. Improving Aboriginal women’s experiences of maternity care: results of the Aboriginal Families Study. Birth. 2015;42(1):27–37. 10.1111/birt.12143 25600655

[9] Buckskin M, Ah Kit J, Glover K, Mitchell A, Miller R, Weetra D, Wiebe J, Yelland J, Newbury J, Robinson J, Brown SJ. Aboriginal Families Study: a population-based study keeping community and policy goals in mind right from the start. Int J Equity Health Care. 2013;12:41. 10.1186/1475-9276-12-41 PMC3689616 23767813

[10] Brown SJ, Yelland JS, Sutherland GA, Baghurst PA, Robinson JS (2011). Stressful life events, social health issues and low birthweight in an Australian population-based birth cohort: challenges and opportunities in antenatal care. BMC Public Health.

[11] https://www.mcri.edu.au/research/projects/aboriginal-families-study (Accessed April 2016)

[12] Australian Institute of Health and Welfare. Measuring the social and emotional well-being of Aboriginal and Torres Strait Islander Health Survey 2004-05. Canberra: Australian Bureau of Statistics; 2006, ABS Cat. No. 4715.0.

[13] Kessler RC, Andrews G, Colpe LJ, Hirilpi E, Mroczek DK, Normand SLT, Walters EE, Zaslav AM. Short screening scales to monitor population prevalences and trends in non-specific psychological distress. Psychol Med. 2002;32:959–76. 10.1017/s0033291702006074 12214795

[14] Kessler RC, Barker PR, Colpe LJ, Epstein JF, Gfoerer JC, Hiripi E, Howes MJ, Normand SLT, Mandersheid RW, Walters EE, Zalavsky AM. Screening for serious mental illness in the general population. Arch Gen Psychiatry. 2003;60:184–9. 10.1001/archpsyc.60.2.184 12578436

[15] Cox JL, Holden JM, Sagovsky R (1987). Detection of postnatal depression: development of the 10-item Edinburgh Postnatal Depression Scale. British J Psychiatr.

[16] Campbell A, Hayes B, Buckby B (2008). Aboriginal and Torres Strait Islander women’s experience when interacting with the Edinburgh Postnatal Depression Scale: a brief note. Aust J Rural Health.

[17] Thomas A, Cairney S, Gunthorpe W, Paradies Y, Sayers S (2010). Strong Souls: development and validation of a culturally appropriate tool for assessment of social and emotional well-being in Indigenous youth. Aust NZ J Psychiatry.

[18] StataCorp (2013). Stata Statistical Software Release 13. College Station.

[19] De Maio JA, Zubrick SR, Silburn SR, Lawrence DM, Mitrou FG, Dalby RB, Blair RM, Griffin J, Milroy H, Cox A (2005). The Western Australian Aboriginal Child Health Survey: Measuring the Social and Emotional Wellbeing of Aboriginal Children and Intergenerational Effects of Forced Separation.

[20] Askew DA, Schluter PJ, Spurling GKP, Bond CJR, Brown ADH (2013). Urban Aboriginal and Torres Strait Islander children’s exposure to stressful events: a cross-sectional study. MJA.

[21] Holt S, Buckley H, Whelan S (2008). The impact of exposure to domestic violence on children and young people: a review of the literature. Child Abuse Negl.

[22] Shonkoff J, Garner A (2012). The lifelong effects of early childhood adversity and toxic stress. Pediatrics.

[23] Glover V (2011). Annual Research Review: Prenatal stress and the origins of psychopathology: an evolutionary perspective. J Child Psychol Psychiatr.

[24] Cker AL, Sanderson M, Dong B (2004). Partner violence during pregnancy and risk of adverse pregnancy outcomes. Paediatr Perinat Epidemiol.

[25] Silverman JG, Decker MR, Reed E, Raj A (2006). Intimate partner violence victimization prior to and during pregnancy among women residing in 26 US states: associations with maternal and neonatal health. Am J Obstet Gynecol.

[26] Sanchez SE, Alva AV, Diez Chang G, Qui C, Yanez D, Gelaye B, Williams MA. Risk of spontaneous preterm birth in relation to maternal exposure to intimate partner violence during pregnancy in Peru. Matern Child Health J. 2013;17(3):485–92. 10.1007/s10995-012-1012-0 PMC3565008 22527763

[27] Shepherd CCJ, Li J, Mitrou F, Zubrick SR (2012). Socio-economic disparities in the mental health of Indigenous children in Western Australia. BMC Public Health.

[28] Yelland J, Sutherland G, Brown SJ (2010). Postpartum anxiety, depression and social health: findings from a population-based survey of Australian women. BMC Public Health.

[29] Buist AE, Austin MP, Hayes BA, Speelman C, Biliszta JL, Gemmill AW, rooks J, Ellwood D, Milgrom J. Postnatal mental health of women giving birth in Australia 2002-2004: findings from the beyondblue National Postnatal Depression Program. Aust NZ J Psychiatry. 2008;42(1):66–73. 10.1080/00048670701732749 18058446

[30] Cunningham J, Paradies Y (2012). Socio-demographic factors and psychological distress in Indigenous and non-Indigenous Australian adults aged 18-64 years: analysis of national survey data. BMC Public Health.

[31] McNamara BJ, Banks E, Gubhaju L, Williamson A, Joshy G, Raphael B, Eades SJ. Measuring psychological distress in older Aboriginal and Torres Strait Islander Australians: a comparison of the K-10 and K-5. Aust NZ J Public Health 2014;online; doi:10.1111/1753-6405.12271 10.1111/1753-6405.12271 25307151

[32] Ekman B, Pathmanathan I, Liljestrand J (2008). Integrating health interventions for women, newborn babies and children; a framework for action. Lancet.

[33] Frenk J (2009). Reinventing primary health care: the need for systems integration. Lancet.

[34] Wong R, Herceg A, Patterson C, Freebairn L, Baker A, Sharp P, Pinnington P, Tongs J. Positive impact of a long-running urban Aboriginal medical service midwifery program. Aust NZ J Obstet Gynaecol. 2011;51:518–22. 10.1111/j.1479-828X.2011.01326.x 21806587

[35] Homer CSE, Foureur M, Allende T, Pekin F, Caplice S, Catling-Paull C (2012). ‘It’s more than just having a baby’ women’s experience of a maternity service for Australian Aboriginal and Torres Strait Islander families. Midwifery.

[36] Murphy E, Best E (2012). The Aboriginal Maternal and Infant Health Service: a decade of achievement in the health of women and babies in NSW. NSW Public Health Bull.

[37] Barclay L, Kruske S, Bar-Zeev S, Steenkamp M, Josif C, Narjic CW, Wardaguga M, Belton S, Gao U, 497 Dunbar T, Kildea S. Improving Aboriginal maternal and infant health services in the ‘Top End’ of Australia; synthesis of the findings of a health services research program aimed at engaging stakeholders, developing research capacity and embedding change. BMC Health Serv Res. 2014;14:241. 10.1186/1472-6963-14-241 PMC4057802 24890910

[38] Di Lallo S (2014). Prenatal care through the eyes of Canadian Aboriginal women. Nurs Womens Health.

[39] Rumbold AR, Cunningham J (2008). A review of the impact of antenatal care for Australian Indigenous women and attempts to strengthen these services. Matern Child Health J.

[40] Jongen C, McCalman J, Bainbridge R, Tsey K (2014). Aboriginal and Torres Strait Islander maternal and child health and wellbeing: a systematic search of programs and services in Australian primary health care settings. BMC Pregnancy Childbirth.

[41] Kramer MS, Séguin L, Lydon J, Goulet L (2000). Socio-economic disparities in pregnancy outcome: why do the poor fare so poorly?. Paediatr Perinatal Epidemiol.

[42] National Insitute of Clinical Excellence (2010). Pregnancy and complex social factors: A model for service provision for pregnant women with complex social factors.

[43] Centre for Maternal and Child Enquiries (CMACE) (2011). Saving Mothers’ Lives: reviewing maternal deaths to make motherhood safer: 2006–08. The Eighth Report on Confidential Enquiries into Maternal Deaths in the United Kingdom. BJOG.

[44] Rayment-Jones H, Murrells T, Sandall J. An investigation of the relationship between the caseload model of midwifery for socially disadvantaged women and childbirth outcomes using routine data – a retrospective, observational study. Midwifery 2015; http://dx.doi.org/10.1016/j.midw.2015.01.003 10.1016/j.midw.2015.01.003 25661044

[45] Mezey G, Bacchus L, Bewley S, White S (2005). Domestic violence, lifetime trauma and psychological health of childbearing women. BJOG.

[46] Hollowell J, Oakley L, Kurinczuk JJ, Brocklehurst P, Gray R (2011). The effectiveness of antenatal care programmes to reduce infant mortality and preterm birth in socially disadvantaged and vulnerable women in high-income countries: a systematic review. BMC Pregnancy Childbirth.

