# Supplementary material for: Stressful events, social health issues and psychological distress in Aboriginal women having a baby in South Australia: implications for antenatal care
Source: BMC Pregnancy Childbirth. 2016 Apr 26;16:88. doi: 10.1186/s12884-016-0867-2 (PMC4845352; doi:10.1186/s12884-016-0867-2)
Supplement: Additional file 1: — STROBE Statement—checklist of items that should be included in reports of observational studies. (DOC 85 kb) [file 12884_2016_867_MOESM1_ESM.doc]

STROBE Statement—checklist of items that should be included in reports of observational studies

|  | Item No | Recommendation |
| --- | --- | --- |
| **Title and abstract** | 1 | (*a*) Indicate the study’s design with a commonly used term in the title or the abstract see page 2 abstract |
|  |  | (*b*) Provide in the abstract an informative and balanced summary of what was done and what was found see page 2 |
| Introduction | | |
| Background/rationale | 2 | Explain the scientific background and rationale for the investigation being reported see pages 3- 4 |
| Objectives | 3 | State specific objectives, including any prespecified hypotheses see page 4 |
| Methods | | |
| Study design | 4 | Present key elements of study design early in the paper see page 4-7 |
| Setting | 5 | Describe the setting, locations, and relevant dates, including periods of recruitment, exposure, follow-up, and data collection, see page 4-5 |
| Participants | 6 | (*a*) *Cohort study*—Give the eligibility criteria, and the sources and methods of selection of participants. Describe methods of follow-up  *Case-control study*—Give the eligibility criteria, and the sources and methods of case ascertainment and control selection. Give the rationale for the choice of cases and controls  *Cross-sectional study*—Give the eligibility criteria, and the sources and methods of selection of participants see page 4-5 |
|  |  | (*b*) *Cohort study*—For matched studies, give matching criteria and number of exposed and unexposed  *Case-control study*—For matched studies, give matching criteria and the number of controls per case |
| Variables | 7 | Clearly define all outcomes, exposures, predictors, potential confounders, and effect modifiers. Give diagnostic criteria, if applicable see pages 5-6 |
| Data sources/ measurement | 8* | For each variable of interest, give sources of data and details of methods of assessment (measurement). Describe comparability of assessment methods if there is more than one group see pages 5-6 |
| Bias | 9 | Describe any efforts to address potential sources of bias See page 8 (assessment of representativeness of sample using routinely collected perinatal data) |
| Study size | 10 | Explain how the study size was arrived at See study protocol, referenced in the paper as reference 9, and provided as supplementary file |
| Quantitative variables | 11 | Explain how quantitative variables were handled in the analyses. If applicable, describe which groupings were chosen and why see pages 5-6 |
| Statistical methods | 12 | (*a*) Describe all statistical methods, including those used to control for confounding see page 6-7 and page 9 (multivariable analyses) |
|  |  | (*b*) Describe any methods used to examine subgroups and interactions |
|  |  | (*c*) Explain how missing data were addressed complete case analysis, few missing data for key exposures or outcome |
|  |  | (*d*) *Cohort study*—If applicable, explain how loss to follow-up was addressed  *Case-control study*—If applicable, explain how matching of cases and controls was addressed  *Cross-sectional study*—If applicable, describe analytical methods taking account of sampling strategy not applicable |
|  |  |  |

| Results | | |
| --- | --- | --- |
| Participants | 13* | (a) Report numbers of individuals at each stage of study—eg numbers potentially eligible, examined for eligibility, confirmed eligible, included in the study, completing follow-up, and analysed - see page 7 |
|  |  | (b) Give reasons for non-participation at each stage see page 7 |
|  |  | (c) Consider use of a flow diagram |
| Descriptive data | 14* | (a) Give characteristics of study participants (eg demographic, clinical, social) and information on exposures and potential confounders See Table 1, and text page 7 |
|  |  | (b) Indicate number of participants with missing data for each variable of interest see Tables 1 and 2 |
|  |  | (c) *Cohort study*—Summarise follow-up time (eg, average and total amount) n/a |
| Outcome data | 15* | *Cohort study*—Report numbers of outcome events or summary measures over time n/a |
|  |  | *Case-control study—*Report numbers in each exposure category, or summary measures of exposure n/a |
|  |  | *Cross-sectional study—*Report numbers of outcome events or summary see Table 3 |
| Main results | 16 | (*a*) Give unadjusted estimates and, if applicable, confounder-adjusted estimates and their precision (eg, 95% confidence interval). Make clear which confounders were adjusted for and why they were included See Tables 1,2, 4 and 6 |
|  |  | (*b*) Report category boundaries when continuous variables were categorized See Tables 1-6 |
|  |  | (*c*) If relevant, consider translating estimates of relative risk into absolute risk for a meaningful time period |
| Other analyses | 17 | Report other analyses done—eg analyses of subgroups and interactions, and sensitivity analyses In addition to analyses reported in tables, we ran additional analyses to account for social support, see page 10 |
| Discussion | | |
| Key results | 18 | Summarise key results with reference to study objectives see page 10-11 |
| Limitations | 19 | Discuss limitations of the study, taking into account sources of potential bias or imprecision. Discuss both direction and magnitude of any potential bias see page 11 |
| Interpretation | 20 | Give a cautious overall interpretation of results considering objectives, limitations, multiplicity of analyses, results from similar studies, and other relevant evidence see pages 12-13 |
| Generalisability | 21 | Discuss the generalisability (external validity) of the study results See page 11 |
| Other information | | |
| Funding | 22 | Give the source of funding and the role of the funders for the present study and, if applicable, for the original study on which the present article is based See page 17 |

*Give information separately for cases and controls in case-control studies and, if applicable, for exposed and unexposed groups in cohort and cross-sectional studies.

**Note:** An Explanation and Elaboration article discusses each checklist item and gives methodological background and published examples of transparent reporting. The STROBE checklist is best used in conjunction with this article (freely available on the Web sites of PLoS Medicine at http://www.plosmedicine.org/, Annals of Internal Medicine at http://www.annals.org/, and Epidemiology at http://www.epidem.com/). Information on the STROBE Initiative is available at www.strobe-statement.org.
